# Detection of COVID-19 Using Transfer Learning and Grad-CAM Visualization on Indigenously Collected X-ray Dataset

**DOI:** 10.3390/s21175813

**Published:** 2021-08-29

**Authors:** Muhammad Umair, Muhammad Shahbaz Khan, Fawad Ahmed, Fatmah Baothman, Fehaid Alqahtani, Muhammad Alian, Jawad Ahmad

**Affiliations:** 1 Department of Electrical Engineering, HITEC University, Taxila 47080, Pakistan; 17-ee-112@student.hitecuni.edu.pk (M.U.); 17-ee-193@student.hitecuni.edu.pk (M.A.); 2 Department of Biomedical Engineering, HITEC University, Taxila 47080, Pakistan; fawad@hitecuni.edu.pk; 3 Faculty of Computing and Information Technology, King Abdul Aziz University, Jeddah 21431, Saudi Arabia; fbaothman@kau.edu.sa; 4 Department of Computer Science, King Fahad Naval Academy, Al Jubail 35512, Saudi Arabia; f-alqahtani@rsnf.gov.sa; 5 School of Computing, Edinburgh Napier University, Edinburgh EH10 5DT, UK; j.ahmad@napier.ac.uk

**Keywords:** COVID-19, artificial intelligence, transfer learning, CNN, X-ray images

## Abstract

The COVID-19 outbreak began in December 2019 and has dreadfully affected our lives since then. More than three million lives have been engulfed by this newest member of the corona virus family. With the emergence of continuously mutating variants of this virus, it is still indispensable to successfully diagnose the virus at early stages. Although the primary technique for the diagnosis is the PCR test, the non-contact methods utilizing the chest radiographs and CT scans are always preferred. Artificial intelligence, in this regard, plays an essential role in the early and accurate detection of COVID-19 using pulmonary images. In this research, a transfer learning technique with fine tuning was utilized for the detection and classification of COVID-19. Four pre-trained models i.e., VGG16, DenseNet-121, ResNet-50, and MobileNet were used. The aforementioned deep neural networks were trained using the dataset (available on Kaggle) of 7232 (COVID-19 and normal) chest X-ray images. An indigenous dataset of 450 chest X-ray images of Pakistani patients was collected and used for testing and prediction purposes. Various important parameters, e.g., recall, specificity, F1-score, precision, loss graphs, and confusion matrices were calculated to validate the accuracy of the models. The achieved accuracies of VGG16, ResNet-50, DenseNet-121, and MobileNet are 83.27%, 92.48%, 96.49%, and 96.48%, respectively. In order to display feature maps that depict the decomposition process of an input image into various filters, a visualization of the intermediate activations is performed. Finally, the Grad-CAM technique was applied to create class-specific heatmap images in order to highlight the features extracted in the X-ray images. Various optimizers were used for error minimization purposes. DenseNet-121 outperformed the other three models in terms of both accuracy and prediction.

## 1. Introduction

The world has been going through an existential pandemic of the COVID-19 disease since December 2019, and it has affected every aspect of our lives [1]. This pandemic has had an intense social and economic impact on most countries. COVID-19 is characterized by a high transmissibility and has a significant mortality rate [2]. All countries have been implementing several precautionary measures to ensure the safety of their citizens. More than 200 countries and territories have been reported to become infected by this virus. As of 29 June 2021, nearly 180 million confirmed cases of COVID-19 and four million deaths have been reported globally [3].

Coronaviruses actually belong to the family of *Coronaviridae*, which is a family of enveloped single-stranded positive-sense RNA viruses [4]. The International Committee on Taxonomy of Viruses named the current discovered novel coronavirus as ‘SARS-CoV-2′, and the disease has been termed ‘COVID-19′ [5,6,7]. SARS-CoV-2 has been listed as one of the novel Betacoronaviruses that infect human beings. It has been investigated that the SARS-CoV-2 is 88% identical to the other two coronaviruses, which were also discovered in China in 2018, i.e., bat-SL-CoVZC45 and bat-SL-CoVZXC21 [8,9]. A study from University College London (UCL) has identified 198 recurring mutations to the virus [10]. The most common symptoms of COVID-19 are a fever, sneezing, cough, sore throat, throat swelling, headache, weakness, malaise, and breathlessness.

The most preferred technique for the diagnosis of COVID-19 is the ‘reverse transcription-polymerase chain reaction’ (RT-PCR) test. However, other non-contact techniques, such as pulmonary X-rays, computed tomography (CT) images, and high-resolution computed tomography (HRCT) images, are also preferred by clinicians [11]. Deep learning and artificial intelligence (AI), in this respect, play a vital role in the accurate detection of COVID-19. In the field of medical data analysis, deep neural networks and AI rapidly gained popularity because they happen to be the most suitable for big data analysis [12]. Deep learning models, especially convolutional neural networks (CNN), are able to automatically learn multiple level of features from data in a hierarchical manner [13]. Furthermore, the interest in the detection of COVID-19 using deep learning and convolutional neural network (CNN) is rapidly increasing.

Recently, quite a few studies have been conducted on the applications of artificial intelligence for the detection and classification of COVID-19. For example, regarding pre-trained models, five pre-trained models, i.e., ResNet-50, ResNet-101, ResNet-152, and Inception-ResNet-v2 were used in [14]. The study uses a dataset of four classes, i.e., ‘COVID’ with 341 images, ‘normal’ with 2800 images, ‘viral pneumonia’ with 1493 images, and ‘bacterial pneumonia’ with 2772 images. However, the pre-trained models have been tested on only two classes at a time for each model, i.e., ‘COVID’ vs. ‘normal’, ‘COVID’ vs. ‘viral pneumonia’, ‘COVID’ vs. ‘bacterial pneumonia’. Similarly, Ref. [15] presents five pre-trained models for three classes in total. The used models are VGG19, MobileNetV2, Inception, Xception, Inception, and ResNet v2. The best performance is achieved on MobileNetV2, with 96.78% accuracy. Furthermore, a computer-aided detection (CAD) was implemented on ResNet-50, Inception V3, DenseNet-201, and Xception in [16]. This study focuses on and presents the division of a highly imbalanced training dataset into a group of small balanced datasets. The use of pre-trained models was also presented in [17], where VGG16, Inception ResNetV2, ResNet-50, Densenet-201, VGG19, MobileNetV2, and NasNet Mobile were utilized. This research utilizes 400 CT scan images and 400 X-ray images, each containing 200 COVID and 200 normal images.

Some of the modified CNN models have also been reported. For example, in [18], a VGG16 based modified CNN was presented, which is named ‘Corona-Net’. The presented model utilizes a three-class classification with two phases, i.e., a re-initialization phase and a classification phase for the detection of COVID-19. Similarly, another modified CNN is presented in [19], in which several parameters have been optimized for the loss minimization purpose. In addition to a training dataset, two external datasets for validation purposes have been utilized. Likewise, VGG16 and VGG19 models have been used in [20] as backbone networks to evaluate the layer depth of the same CNN architecture. Each backbone network has been trained and evaluated with different degrees of fine tuning. In addition to the aforementioned modified CNNs, a modified inception model is presented in [21], where 1065 CT scan images were used. The presented accuracy is 89.5%. A multi-view fusion deep learning network based on ResNet-50 is presented in [22]. A total of 368 COVID-19 and 127 pneumonia images were used. The idea is to train the model in multi-view images of chest CT images, which improves the efficacy of the diagnosis.

Some other methods have also been reported, and one such model is presented in [23]. This study utilized Inception-ResNet-v2 as the based model. The presented model is named ‘CoVIR-Net’. They exhibited two approaches: CoVIR-Net with Inception-Resnet-v2 residual blocks and the CoVIR-Net feature extractor with a random forest classifier. The two approaches presented accuracies of 95.78% and 97.29%, respectively. Another method named by the authors as ‘COVID-Net’ is presented in [24]. The presented architecture utilizes a lightweight residual PEPX (projection-expansion-projection-extension) design pattern. The presented article utilizes an open-source dataset, COVIDx. This dataset has 13,975 X-ray images that have been collected from 13,870 patient cases. The presented model reports a 93.3% test accuracy. Similarly, a ResNet-50-based model termed ‘DREnet’ is presented in [25]. It was used for the diagnosis of CT images that were divided into three classes. The achieved accuracy is reported as 89.36%. A modified model named ‘DeCoVNet’ has been presented in [26], which use UNet as the base model. The presented model was tested on 499 CT scan images, with an accuracy of 95.9%. In addition, a model called ‘DarkCovidNet’ is presented in [27], in which the DarkNet model has been utilized as a classifier for the YOLO real time object selection. It exhibited a binary and multiclass accuracy of 98.08% and 87.02%, respectively. Another model, named ‘Coronet’, is presented in [28], with the use of Xception as the base model. A total of 203 normal and 660 bacterial pneumonia images were used. The reported accuracy is 89.6%.

A comparative study was performed in [29] using ResNet-18, ResNet-50, COVID-NET, and DenseNet-121 on Pytorch 1.4. The collection of a custom dataset was reported (named as ‘CORDA’), which consists of data of 386 patients from a hospital in Turin, Italy. Six other publicly available datasets were also utilized. In addition to the implementation of the aforementioned models, authors have also used a new convolutional neural network comprising of eight convolution layers and a fully connected layer. This new model has been named ’Conv8’. The use of this smaller architecture did not perform well in comparison to large models, such as ResNet-18, but presented acceptable results, with a BA of 0.61 and DOR of 2.38. The main constraint of the study was the lack of availability of a large dataset. The authors suggested that these models can perform quite well upon the availability of a larger dataset. In comparison, the re-search work in this paper focuses on the use of fine tuning and transfer learning by utilizing the pre-trained weights through freezing the pre-trained layers. Only the weights of the last two layers have been re-trained/updated. Furthermore, a large dataset of 7232 images has been used in this research, which improves the accuracies of the models used in this paper. Moreover, the testing dataset has been segregated from the training and validation datasets. Apart from this segregated dataset, a locally collected dataset of 450 images has also been utilized for the purpose of testing and prediction.

In this research, the transfer learning technique is used to detect COVID-19 from the pulmonary (chest) X-rays. For this purpose, four pre-trained CNN models, i.e., VGG16, ResNet50, MobileNet, and DenseNet-121 have been used. In order to utilize both the transfer learning technique and the fine-tuning on the aforementioned pre-trained models, batch normalization and dropout layers are added in the FC layer. Details on the implementation of transfer learning are given in Section 2.3. The models under study have been compared on the basis of important parameters, such as the number of epochs, batch size, learning rate, etc. Suggestions have been made on the basis of comparison and critical analysis. The models have been tested on 450 indigenously collected X-ray images of Pakistani patients. Finally, the Grad-CAM technique has been applied in order to create class-specific heatmap images to highlight the features that are extracted from the X-ray images. Various optimizers, i.e., Adam, the stochastic gradient descent (SGD), Adadelta, and RMSprop from Keras version 2.6.0 have been used for error minimization purpose. It is noteworthy to mention here that the pretrained models had originally been trained on the Imagenet dataset, which consisted of millions of images with multiple classes. Whereas, in this research, these models have been retrained on 5062 images assessed through the Kaggle website for two classes only. By utilizing transfer learning and fine tuning, a higher accuracy is achieved for each model. Tensorflow and Keras API is used for the processing of these models. This study was performed on a 12 GB NVIDIA Tesla K80 GPU that was provided online by Google Colab, a product of Google Research headquartered at Mountain View, CA, United States.

## 2. Materials and Methods

### 2.1. Dataset

**Figure 1:**
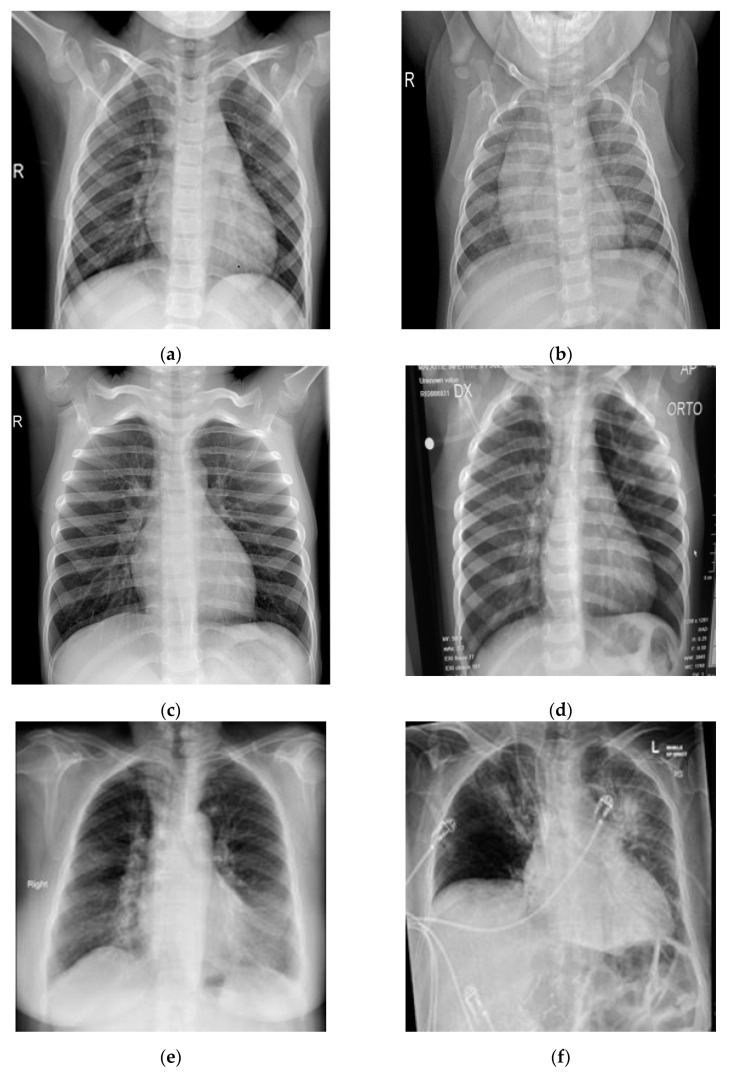
Samples of chest X-ray images assessed via Kaggle database [30,31,32] for training purposes: (**a**–**c**) normal chest X-rays; (**d**–**f**) COVID-19 chest X-rays.

**Figure 2:**
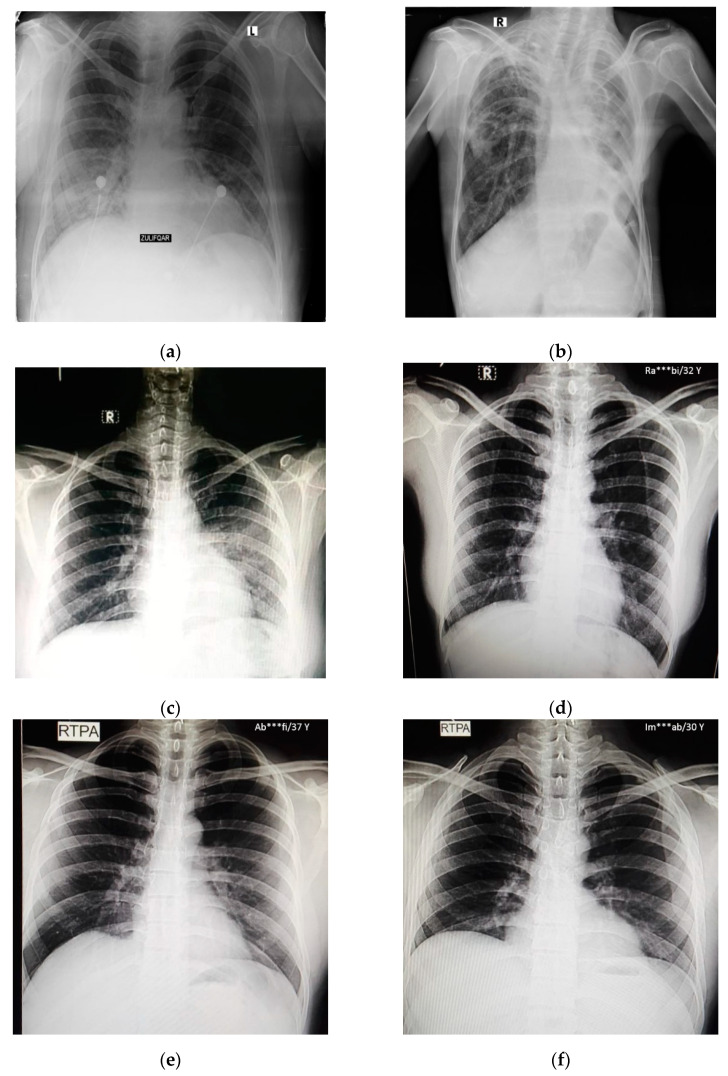
Samples of local Pakistani verified dataset used for the testing of trained models: (**a**–**c**) COVID-19 chest X-rays; (**d**–**f**) normal chest X-rays.

In this study, pulmonary (chest) X-ray images were used for the diagnosis of COVID-19. The dataset was categorized into two main classes, i.e., COVID-19 and normal. A total of 7232 images (3616 COVID-19 + 3616 normal) were accessed from the ‘COVID-19 Radiography Database (available at Kaggle, https://www.kaggle.com/tawsifurrahman/covid19-radiography-database, accessed on 4 May 2021) [30]. From the total images, 70% (5062 images) were used for training + validation, and the remaining 30% (2170 images) were used for testing purposes. The training + validation dataset of 5062 images was further split into a 70:30 ratio, i.e., 3544 (70%) images for training purposes and 1518 (30%) images for validation purposes. The details of the data splitting are given in Table 1. In addition to the dataset accessed via Kaggle, another locally collected dataset of 450 images (COVID-19 + normal) was also used for testing and prediction purposes. This indigenous data of chest X-ray images of Pakistani COVID-19 positive and normal patients have been collected from a local hospital. The samples of normal and COVID-19 X-ray images assessed via the Kaggle database are shown in Figure 1, whereas the samples of the locally collected images are given in Figure 2.

### 2.2. Methodology

**Figure 3:**
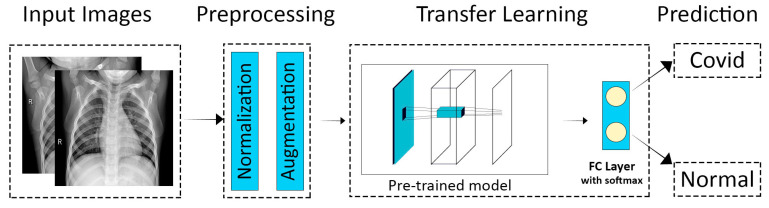
Overview of the transfer learning technique for the diagnosis of COVID-19 [33].

**Figure 4:**
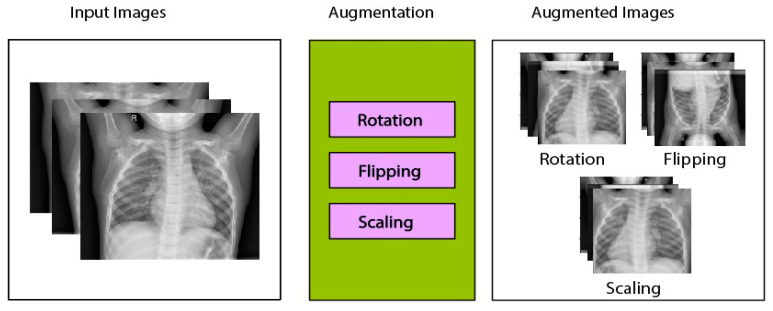
Augmentation techniques applied on input images used in this study [33].

**Figure 5:**
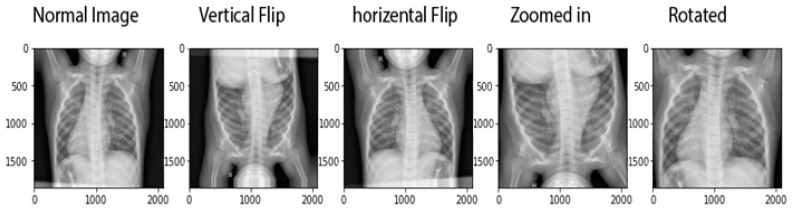
Samples of data augmentation techniques applied on a single image.

The methodology adopted for transfer learning for the detection and classification of COVID-19 is depicted in Figure 3. The primary objective was to classify a chest X-ray image into two categories, i.e., normal and COVID-19. The two main stages involved in the model were the preprocessing stage (which further included normalization and data augmentation) and the classification stage (which involved the use of transfer learning on pre-trained models and prediction). A normalization range of 0 to 1 was used. The images were rescaled by multiplying each pixel with a factor of 1/255. Moreover, the images were augmented by the following: (1) rotation at 40 degrees, (2) height, width, and zoom range scaling, and (3) horizontal flipping and vertical flipping. The data augmentation performed on the training images is shown in Figure 4. A sample of the results obtained after the data augmentation is depicted in Figure 5.

**Figure 6:**
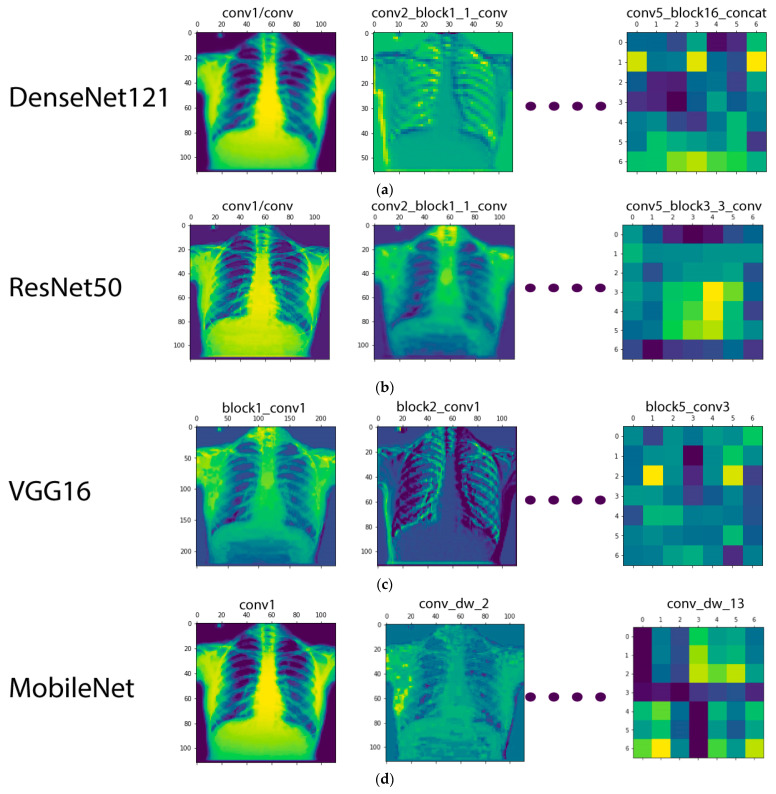
Visualization of the feature maps for the CNN models used in this study: (**a**) DenseNet-121; (**b**) ResNet-50; (**c**) VGG16; (**d**) MobileNet.

During the training process in a CNN model, the visualization of intermediate activations helps to better understand the feature extraction process, especially for an image-based dataset. The term activation here is referred to as the output of a layer, or, more specifically, the outputs of the several pooling and convolution layers are termed ‘feature maps’. Therefore, the purpose of visualizing the intermediate activations was actually to display these feature maps in order to better understand the process of decomposition of an input image into various filters learned by the network. The visualization of the intermediate activations for the CNNs used in this study is given in Figure 6.

### 2.3. Architecutral Overview of Pre-Trained Models

**Figure 7:**
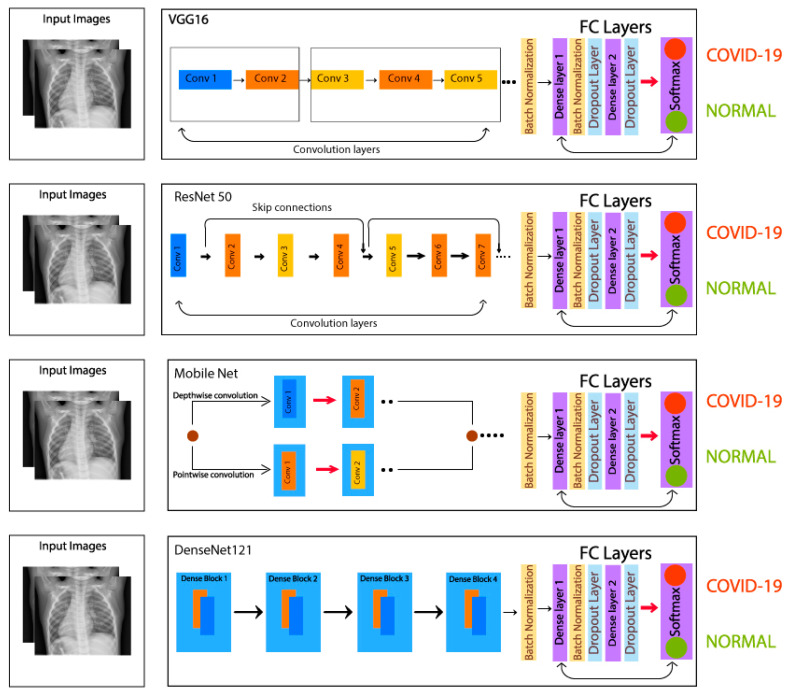
Overview of the architecture of four CNN models used in this research.

The VGG16 contains 13 convolutional layers and 3 fully connected dense layers. The ResNet-50 contains 50 layers, and skip connection technique is used in this residual network, which skips convolutional layers that help a lot during backpropagation method. The original model consists of 16 residual blocks and one dense layer, whereas, in this study, three dense layers are added and utilized. Regarding MobileNet, it is a lightweight neural network with lesser parameters and higher image classification accuracy. MobileNet contains 28 convolutional layers and comprises the depthwise separable convolution. The depthwise separable convolution further contains two layers, i.e., depthwise convolution, and pointwise convolution. DenseNet-121, on the other hand, contains 121 layers and has 4 dense blocks. The architectural parameters of the four models under study are given in Table 2, and the architectural designs of the models utilized for transfer learning are shown in Figure 7.

The models used in this research have already been trained on a large scale labeled dataset, i.e., ImageNet. During the training phase, the pre-trained weights (the weights obtained when a model was trained on the ImageNet dataset) were utilized. Transfer learning was applied by freezing the pre-trained layers, except for the last two layers of each model. The last two layers were unfrozen and were retrained on our dataset. Moreover, a batch normalization layer was added before the fully connected layer in each model. After flattening the model in the fully connected layer, dense layer 1 was added, which contains 4096 units with ReLU activation function. A batch normalization layer was added again after the dense layer 1. After the batch normalization layer, a dropout layer was added with a dropout size of 0.5. Then, a dense layer 2 was added, which contains 1024 units with the activation function ReLU. Another dropout layer was then added after the dense layer 2, with a dropout size of 0.5, and, finally, a dense layer 3 was added, which contains 2 units with the Softmax activation function.

Usually, a great number of data are needed to train a neural network from scratch, but access to data is not always available, e.g., the COVID-19 radiography data. Utilizing a pretrained model with pretrained weights addresses the issue of large training data requirement. The learning process becomes faster and more accurate and needs less training data. Traditional learning, in comparison, starts with randomized weights and tunes them until they finally converge. Transfer learning, on the other hand, offers a higher learning rate during training. Therefore, with a better starting point and a higher learning rate, transfer learning converges the neural networks faster and at a higher performance level, enabling more accurate outputs.

In this study, fully connected (FC) layers, batch normalization layers, and a dropout of 0.5 was added to the model for the purpose of fine tuning. Details pertaining to the trainable and non-trainable parameters are given in Table 3. The FC layer was kept the same for all of the models. In the FC layer, the dense layer 1 and dense layer 2, an activation function of ‘ReLU’ with 4096 and 1024 units was used, respectively. Whereas, in the dense layer 3, two units, along with the ‘softmax’ activation function, was used. This activation function gave us the output from the 2 classes, i.e., COVID-19 and normal. Even though, for binary classification, a simple Sigmoid can also work, in comparison, the Softmax as an output layer works better. The probabilities produced by Sigmoid do not sum up to the value of 1. Whereas, the output probabilities of Softmax are interrelated and always sum up to the value 1. In case of Softmax, increasing the output value of one class makes the output of other class go down. Furthermore, while using ReLU as the activation function, use of Softmax as the output layer is mostly preferred [34]. In a recent research [34], the authors evaluated the performance capability of the Softmax output layer with the ReLU activation layer for several neural networks and validated better performance of Softmax with ReLU activation layers for classification tasks. Hence, Softmax as an output layer in this research is primarily preferred in order to obtain prediction in the form of probabilities. As for the purpose of predicting the final result, it is convenient to know how much our model is closer to predicting the specific class. In addition, in order to have a high probability for one class, the probability of the other class has to decrease by an equal amount; thus, Softmax works better with ReLU activation layers [34].

In addition to dense layers, batch normalization layers were used, which help to converge the model faster and achieve lower error in the training phase. The size of input images was kept constant to 224 × 224 pixels for all four models. However, for the purpose of fine tuning, the last two layers of the pre-trained model were unfrozen and trained on the training dataset used in this study. The final architectures used for transfer learning are given in Figure 7.

### 2.4. The Grad-CAM Technique

**Figure 8:**
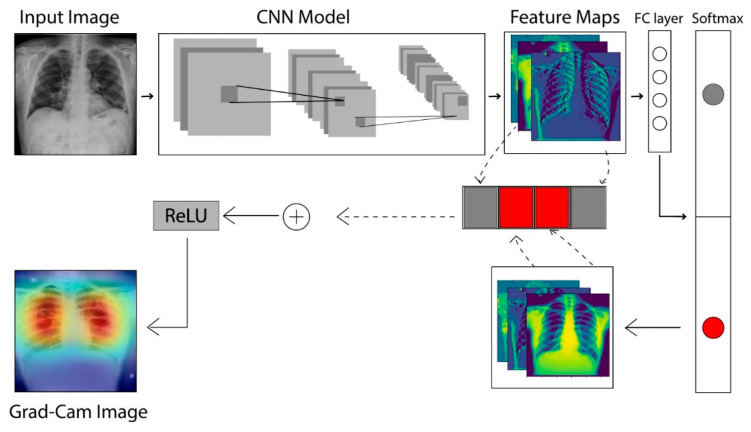
Architecture to describe the Grad-CAM technique [36].

In CNNs, the gradient class activation map (Grad-CAM) is a technique that is used to create a class-specific heatmap. This generated class-specific heatmap is based on a specific input image using a trained CNN model [35]. The Grad-CAM technique is employed to figure out the COVID-19 detection transparency. This technique actually highlights the regions of the input image, which is where the model pays much attention during the classification process, implying the fact that the feature maps generated in the final convolution layer hold the spatial information that helps in capturing the visual pattern. This visual pattern contributes in distinguishing assigned classes. The Grad-CAM technique is applied by utilizing the layers and extracted features of the trained model. The architecture explaining the Grad-CAM technique is shown in Figure 8.

**Figure 9:**
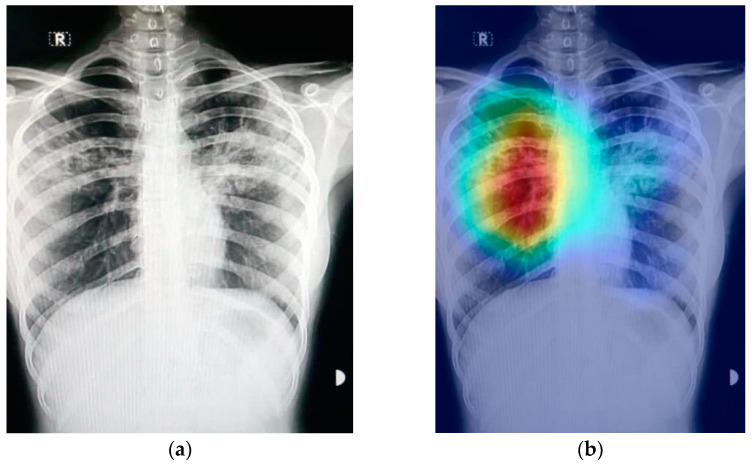
Grad-CAM technique results on the COVID-19 X-ray image: (**a**) COVID-19 X-ray image; (**b**) Grad-CAM of the image.

Figure 9 shows an example of a chest X-ray image generated using the Grad-CAM technique. The original chest X-ray image is shown in Figure 9a, whereas the overlay heatmap on the input image is shown in Figure 9b. The jet color scheme is used in this study. In this color scheme, blue tones represent lower values, which means that no features are extracted for a specific class, whereas the yellow and green tones represent medium values depicting quite less feature extraction, and the red and dark red tones represent larger values, i.e., the features in the region represent the specific class.

## 3. Implementation and Results

### 3.1. Implementation Details and Performance Parameters

The prepared dataset is evaluated using the four pre-trained models, i.e., VGG16, ResNet-50, DenseNet-121, and MobileNet. A 70:30 training–validation ratio was utilized, and the data splitting details can be found in Table 1. The training of the four models in this study was performed on the augmented data. For training, the dataset images were resized to 224 × 224 pixels. Besides, a batch size of 32 is kept with the number of epochs, which is 80. These values were finalized using the grid search approach. The learning rate was fixed to 0.0001 for the training of each model.

Furthermore, in order to evaluate the performance of each model, the important performance parameters, i.e., precision, recall, specificity, accuracy, and F1-score were calculated. These parameters were calculated using Equations (1)–(5). The quantities involved in the calculation of aforementioned performance parameters, i.e., True Positive (*TP*), True Negative (*TN*), False Positive (*FP*), and False Negative (*FN*) were obtained from confusion matrices [37].

(1) $$Precision=\frac{nTP}{nTP+nFP}$$

(2) $$Recall=\frac{nTP}{nFN+nTP}$$

(3) $$Specificity=\frac{nTN}{nFP+nTN}$$

(4) $$Accuracy=\frac{nTP+nTN}{nFP+nTP+nTN+nFN}$$

(5) $$F1-score=2\times \frac{Precision\times Recall\;}{Precision+Recall}$$

The normal and COVID-19 are considered as negative and positive cases, respectively. Therefore, 𝑛*TN* and 𝑛*TP* represent the correctly predicted normal and correctly predicted COVID-19 images, respectively, whereas 𝑛*FN* and 𝑛*FP* represent the falsely predicted normal and falsely predicted COVID-19 cases, respectively.

### 3.2. Results and Discussion

**Figure 10:**
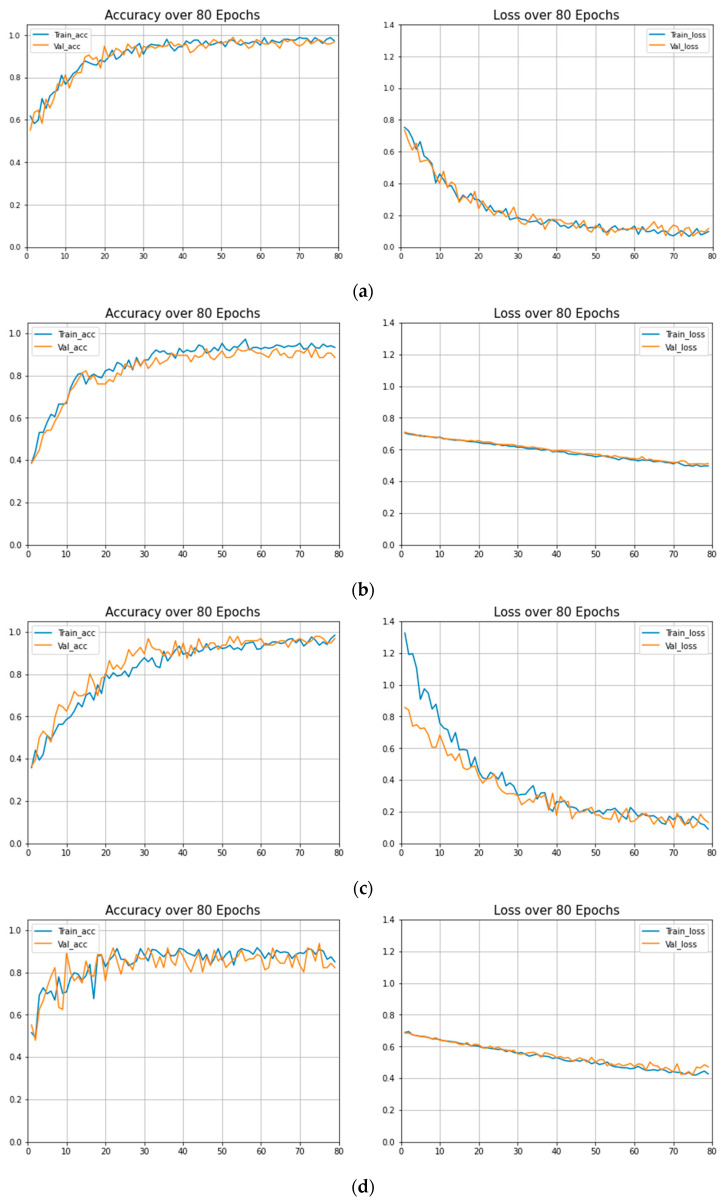
Training accuracies vs. validation accuracies and training loss vs. validation loss: (**a**) MobileNet; (**b**) ResNet-50; (**c**) DenseNet; (**d**) VGG16.

The training performance of the models under study were evaluated in terms of important parameters, i.e., training accuracy, validation accuracy, training loss, and validation loss at different epochs. The results of these parameters can be found in Table 4. These parameters are calculated to estimate the over-fitting and under fitting of the trained models. The graphs of training loss vs. validation loss and training accuracy vs. validation accuracy of each model are given in Figure 10. The training curves are reported on a population of 3544 patients from the training dataset, whereas the validation curves are reported on a population of 1518 patients from the validation dataset. Details of the training and validation datasets with data splitting ratios are given in Section 2.1 and Table 1. It can be observed that the Dense-Net-121 has the minimum training and validation loss and exhibits the best training and validation accuracy.

**Figure 11:**
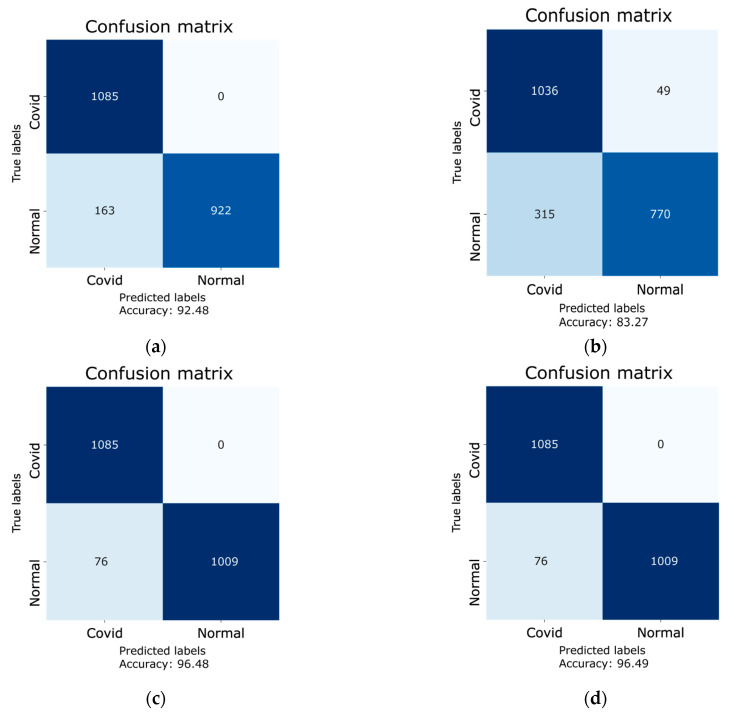
Confusion matrices of each model for the results on the testing dataset: (**a**) ResNet-50 model; (**b**) VGG16; (**c**) MobileNet; (**d**) DenseNet-121.

Furthermore, confusion matrices for all four models were generated in order to quantify the performance metrics, i.e., precision, F1-score, recall, specificity, and accuracy. The results of the aforementioned parameters are given in Table 5. The confusion matrices were generated using the testing dataset of 2170 images. These images were not included in the training and validation datasets. The confusion matrices of each model is given in Figure 11, whereas the true labels and predicted labels from confusion matrices with accuracies and 95% confidence intervals (CI) are presented in Table 6. In addition to calculating the confidence intervals, the Cohen’s kappa coefficient was also calculated for each model, which confirms the reliability of the implemented models. The DenseNet-121 has an accuracy of 96.49% (2094/2170), with a 95% confidence interval of [0.96, 0.97], and the computed Cohen’s kappa coefficient for Dense-Net-121 is 0.92. The ResNet-50 has an accuracy of 92.48% (2007/2170), with a 95% confidence interval of [0.91, 0.94], and the computed Cohen’s kappa coefficient for Res-Net-50 is 0.85. The VGG16, on the other hand, has an accuracy of 83.27% (1806/2170), with a 95% confidence interval and Cohen’s kappa coefficient of [0.82, 0.85] and 0.66, respectively. Besides, the MobileNet model has an accuracy of 96.48% (2094/2170), with a 95% confidence interval of [0.96, 0.97] and a Cohen’s kappa coefficient of 0.92. The result of the Cohen’s kappa coefficient lying in a range of 0.61–0.80 is considered to be a substantial agreement with the presented results, whereas the range of 0.81–1.00 is considered as an almost perfect agreement with the presented results [37,38]. It is evident from the aforementioned results that the Cohen’s kappa coefficients of the three retrained models, i.e., DenseNet-121, Resnet-50, and MobileNet, lie in the range of 0.81–1.00, and that the Cohen’s kappa coefficient of the VGG16 model lies in the range of 0.61–0.80. This confirms the reliability of the implemented models. 

Using the numbers obtained via the confusion matrices, the performance parameters for all four models were calculated using Equations (1)–(5). The results of these parameters are given in Table 5. It can be seen that among the four models under study, the DenseNet-121 shows an eminent performance with a recall of 100%, specificity of 92.99%, F1-score of 0.97, and accuracy of 96.49%. When benchmarked with the popular convolutional neural network COVID-Net, which has an accuracy of 93.3%, two of the four retrained models in this research, i.e., DenseNet-121 and MobileNet exhibit an improved accuracy of 96.49% and 96.48%, respectively.

#### 3.2.1. Optimizers

Four optimizers, i.e., SGD, Adadelta, RMSprop, and Adam were applied on the two best trained models in order to compare the performance of the optimizers and to select on optimizer which can be applied on all four models under study. The parameters obtained for Densenet-121 and MobileNet after applying the four aforementioned optimizers are given in Table 7. It can be seen that the RMSprop optimizer exhibited promising results among all four optimizers. Hence, the RMSprop optimizer was chosen to be applied on the four trained models. The results previously shown in Table 5 are actually RMSprop-optimized results.

#### 3.2.2. Learning Rate

**Figure 12:**
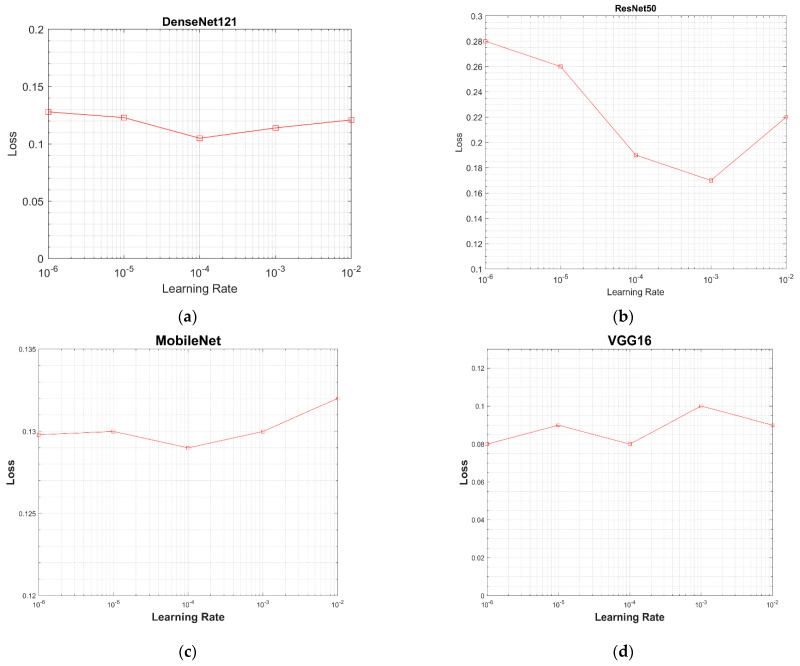
Graphs between testing loss and learning rate for all models: (**a**) loss vs. learning rate graph of DenseNet-121; (**b**) loss vs. learning rate graph of ResNet-50; (**c**) loss vs. learning rate graph of MobileNet; (**d**) loss vs. learning rate graph of VGG16.

In order to choose an optimum learning rate for all models, the models were evaluated over a selected range of learning rates, i.e., from 10^−2^ to 10^−6^ (0.01, 0.001, 0.0001, 0.00001, 0.000001). The value of the testing loss was calculated for each model using the testing dataset of 2170 images (dataset details have been provided in Section 2.1 and Table 1). A value of the learning rate at the minimum loss was considered best for a model. The graphs between learning rates and values of the testing loss are given in Figure 12. In Figure 12a, the DenseNet-121 has the minimum testing loss of 0.1 at the learning rate of 0.0001. ResNet-50 has the minimum testing loss of 0.15 at 0.01, as shown in Figure 12b. MobileNet has the minimum testing loss of 0.129 at a 0.0001 learning rate, as shown in Figure 12c, and VGG16 has the minimum testing loss of 0.08 at a 0.0001 learning rate, as well as the same testing loss at a 0.00001 learning rate, as shown in Figure 12d. A learning rate of 0.0001 was chosen based on these results.

#### 3.2.3. Optimal Batch Size

In the training of a CNN model, the batch size plays an indispensable role. The role of the batch size in the improvement of the testing accuracy is studied. The values of test accuracies based on different batch sizes, i.e., 8, 16, and 32 are given in Table 8. It is evident that, for a batch size of 32, all of the models demonstrate highest test accuracies. Therefore, a batch size of 32 was set for this work.

#### 3.2.4. Prediction

By applying the transfer learning technique on the pre-trained models, we trained the augmented chest X-ray images, whereas for the testing and validation, local verified images of COVID-19-positive and normal patients were used. The prediction of ResNet-50, MobileNet, and Densenet-121 was accurate in determining whether the X-ray under test is of a COVID-19 patient or of a normal patient. However, VGG16 displayed the least accurate prediction and misclassified the test images because of its low testing accuracy and loss.

**Figure 13:**
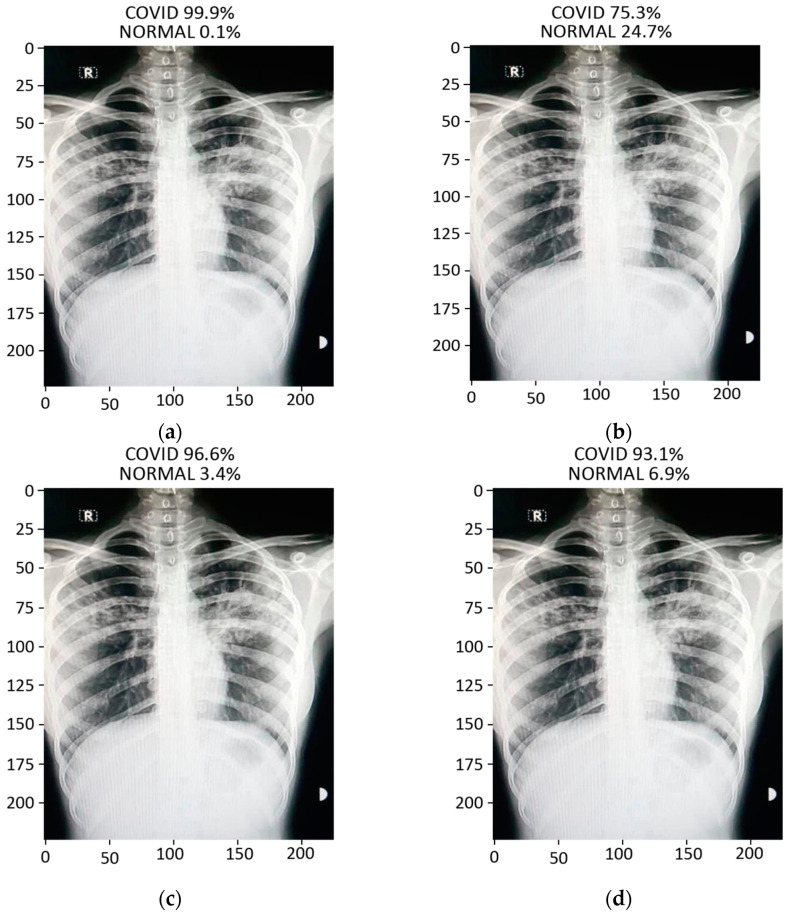
Results of prediction on a verified local Pakistani COVID-19 chest X-ray: (**a**) DenseNet-121; (**b**) VGG16; (**c**) MobileNet; (**d**) ResNet-50.

**Figure 14:**
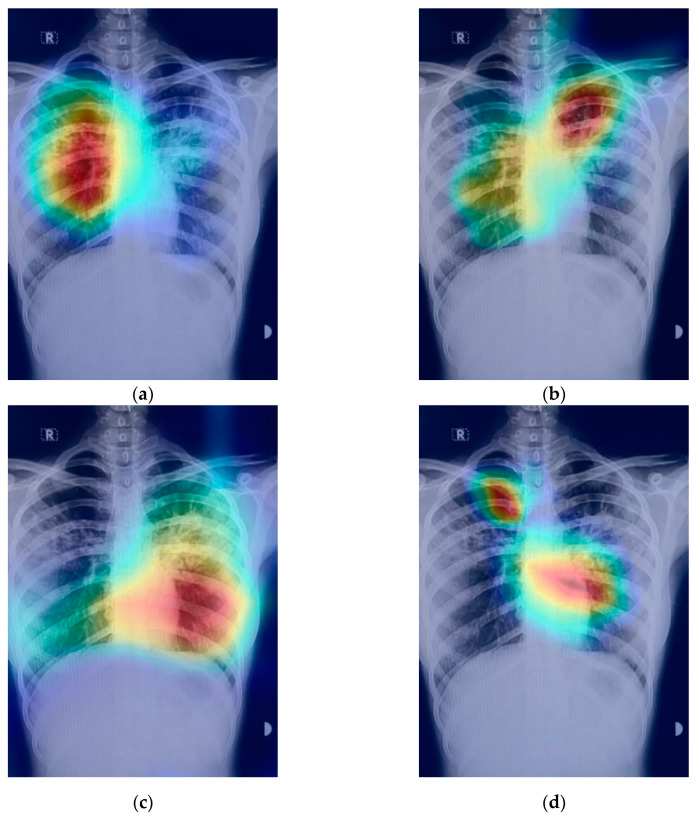
Grad-CAM results on local Pakistani COVID-19 chest X-ray: (**a**) DenseNet-121; (**b**) VGG16; (**c**) MobileNet; (**d**) ResNet-50.

**Figure 15:**
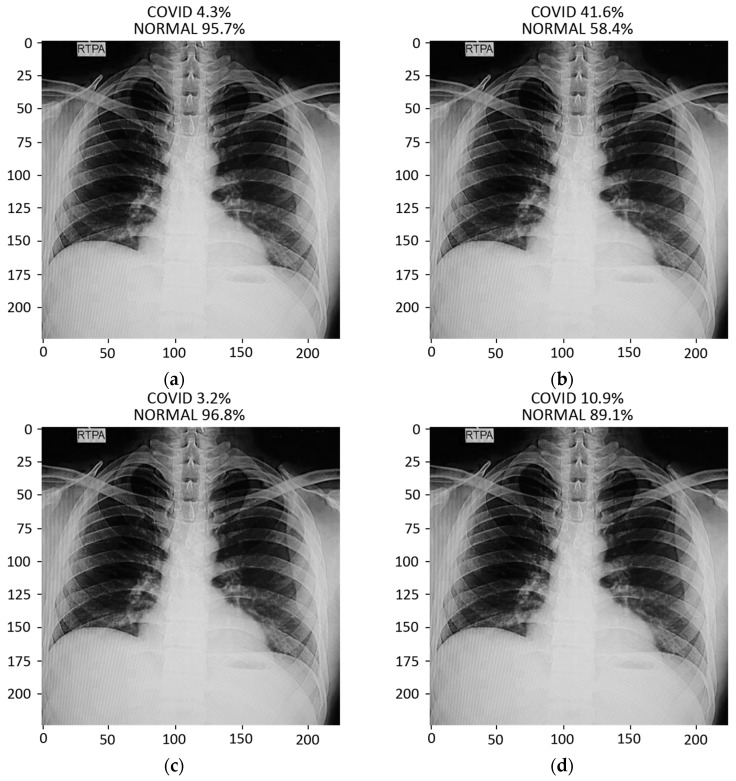
Results of prediction on a verified local Pakistani normal chest X-ray: (**a**) DenseNet-121; (**b**) VGG16; (**c**) MobileNet; (**d**) ResNet-50.

The prediction result for a chest X-ray of a local COVID-19-positive patient for each model is given in Figure 13a–d. The DenseNet-121 had the highest probability percentage of COVID-19, i.e., 99.9%. In order to highlight the area where the model is paying the most attention during feature extraction, the Grad-CAM technique was applied. The Grad-CAM results of all four models under study are given in Figure 14. Moreover, the results of the predicted normal patients for each model are shown in Figure 15a–d. Considering these results, the MobileNet has the highest probability of 96.8% for the prediction of a normal chest X-ray.

## 4. Conclusions

In this paper, we applied transfer learning technique with fine tuning on the four pre-trained models (VGG16, ResNet-50, MobileNet, and DenseNet-121) in order to detect COVID-19 using chest X-ray images acquired from a hospital in Pakistan. The models under study were trained using the dataset of more than 3600 COVID-19 and normal chest X-ray images, whereas an indigenously collected dataset of 450 X-ray images of Pakistani patients were used for testing and prediction purposes. Various important parameters, e.g., recall, specificity, F1-score, precision, loss graphs, and confusion matrices were used to validate the accuracy of the models. The VGG16 model exhibited the least accurate performance in classifying the COVID-19 and normal chest X-ray images; however, the DenseNet-121 displayed promising results in the classification of the COVID-19 and normal images. The achieved accuracies of VGG16, ResNet-50, DenseNet-121, and MobileNet are 83.27%, 92.48%, 96.49%, and 96.48%, respectively. Furthermore, in order to highlight the area where the model is paying the most attention during feature extraction, the Grad-CAM technique was also applied to create class-specific heatmap images. Various optimizers were tested, and among all of the optimizers under study, the ‘RMSprop’ optimizer exhibited the best performance and hence was applied for the error minimization and better optimization in the training procedure.

## Figures and Tables

**Table 1:** Details of data splitting.

Classes	Dataset			
	Training	Validation	Testing	Total
COVID-19	1772	759	1085	3616
Normal	1772	759	1085	3616
Total	3544	1518	2170	7232

**Table 2:** Architectural parameters of the four CNN models used in this research.

Models	Layers	Input Layer Size	Output Layer Size
ResNet-50	50	(224, 224, 3)	(2, 1)
VGG16	16	(224, 224, 3)	(2, 1)
MobileNet	28	(224, 224, 3)	(2, 1)
DenseNet-121	121	(224, 224, 3)	(2, 1)

**Table 3:** Parameters of the pre-trained models in this study.

Models	Total Parameters	Trainable Parameters	Non-Trainable Parameters
ResNet-50	439,027,730	415,440,018	23,587,712
VGG16	121,873,362	107,158,674	14,714,688
MobileNet	213,147,986	209,919,122	3,228,864
DenseNet-121	216,956,626	209,921,170	7,035,456

**Table 4:** Training performance of the CNN models used in this work.

Models	Epochs	Training Loss	Validation Loss	Training Accuracy	Validation Accuracy
MobileNet	1	0.8837	0.7963	51.44%	52.08%
	.	.	.	.	.
	.	.	.	.	.
	79	0.0721	0.0954	98.92%	95.83%
	80	0.0797	0.1171	98.03%	96.88%
VGG16	1	0.7002	0.7041	53.94%	48.96%
	.	.	.	.	
	.	.	.	.	
	79	0.4453	0.4850	87.40%	84.38%
	80	0.4272	0.4723	85.04%	82.29%
DenseNet-121	1	1.3241	0.8617	39.67%	39.81%
	.	.	.	.	
	.	.	.	.	
	79	0.0952	0.1587	97.92%	96.73%
	80	0.0583	0.0617	98.96%	97.13%
ResNet-50	1	0.7008	1.1915	44.74%	39.58%
	.	.	.	.	
	.	.	.	.	
	79	0.4974	0.5083	93.75%	90.62%
	80	0.4911	0.5123	92.08%	88.54%

**Table 5:** Comparison of performance parameters of CNN models.

Model	Pre (%)	F1-Score	Recall (%)	Spe (%)	Acc (%)
MobileNet	86.93	0.97	100	92.99	96.48
VGG16	76.80	0.85	95.51	70.96	83.27
DenseNet-121	93.45	0.97	100	92.99	96.49
ResNet-50	86.93	0.93	100	84.97	92.48

**Table 6:** Confusion matrix of models with accuracies and 95% confidence intervals.

Models	True Labels	Predicted Labels		Accuracy	95% CI
		COVID-19	Normal		
DenseNet-121	COVID-19	1085	0	96.49%	[0.96, 0.97]
	Normal	76	1009		
ResNet-50	COVID-19	1085	0	92.48%	[0.91, 0.94]
	Normal	163	922		
VGG16	COVID-19	1036	49	83.27%	[0.82, 0.85]
	Normal	315	770		
MobileNet	COVID-19	1085	0	96.48%	[0.96, 0.97]
	Normal	76	1009		

**Table 7:** Comparison of classification performance of DenseNet-121 and MobileNet among different optimizers.

Models	Optimizers	Pre %	Recall %	Spe. %	F1-Score	Acc. %
DenseNet-121	SGD	86.09	92.99	84.97	0.89	88.98
	Adadelta	95.52	88.47	89.27	0.91	93.16
	Adam	91.47	94.93	96.7	0.93	93.50
	RMSprop	93.45	100	92.9	0.97	96.49
MobileNet	SGD	98.51	91.98	98.61	0.95	95.29
	Adadelta	96.98	71.15	97.78	0.82	84.47
	Adam	92.38	97.23	91.98	0.95	94.07
	RMSprop	93.45	100	92.9	0.97	96.48

**Table 8:** Test accuracies based on batch sizes.

Models	Batch Size		
	8	16	32
VGG16	85.67%	83.67%	83.27%
ResNet-50	89.94%	89.95%	92.48%
MobileNet	94.5%	95.0%	96.48%
DenseNet-121	92.67%	95.83%	96.49%
